# Economic evidence of preventive interventions for anxiety disorders in children and adolescents – a systematic review

**DOI:** 10.1192/j.eurpsy.2022.1761

**Published:** 2022-09-01

**Authors:** A.-K. Vartiainen, V. Kuvaja-Köllner, M. Rantsi, E. Rissanen, T. Luntamo, M. Kurki, A. Sourander, E. Kankaanpää

**Affiliations:** 1 University of Eastern Finland, Department Of Health And Social Management, Kuopio, Finland; 2 University of Turku, Research Center For Child Psychiatry, Turku, Finland

**Keywords:** prevention, Anxiety, economic evaluation, children and adolescents

## Abstract

**Introduction:**

Anxiety disorders are common in children and youth. Also, in prevention, be it universal, selective or indicated, economic evaluation supports decision-making in the allocation of scarce resources.

**Objectives:**

This review identified and summarised the existing evidence of economic evaluations for the prevention of anxiety disorders in children and adolescents.

**Methods:**

A systematic search was conducted on the EBSCO, Scopus, Web of Science, ProQuest, Cochrane and PubMed databases. We included studies that focused on children and adolescents under 18 years of age, aimed to prevent anxiety disorders, and presented an incremental analysis of costs and effectiveness. A registered checklist was used that assessed the quality of the included articles.

**Results:**

The search yielded 1,697 articles. Five articles were included in this review. Three were RCT-based and two were model-based studies. Out of five included interventions, one was a universal school-based intervention, two selective interventions and two indicated interventions. Universal school-based prevention of anxiety was not cost-effective compared to usual teaching. Selective parent training and indicative child- and parent-focused CBT prevention were likely cost-effective compared to usual care or doing nothing.

**Conclusions:**

Parent education and cognitive behaviour therapy interventions can be cautiously interpreted as being a cost-effective way of preventing anxiety in children and adolescents. However, the evidence is weak related to cost-effectiveness as there are only a few studies, with relatively small sample sizes and short follow-ups.

**Disclosure:**

No significant relationships.

